# Machine learning outperforms thermodynamics in measuring how well a many-body system learns a drive

**DOI:** 10.1038/s41598-021-88311-7

**Published:** 2021-04-29

**Authors:** Weishun Zhong, Jacob M. Gold, Sarah Marzen, Jeremy L. England, Nicole Yunger Halpern

**Affiliations:** 1 Physics of Living Systems, Department of Physics, Massachusetts Institute of Technology, 400 Tech Square, Cambridge, MA 02139 USA; 2 Department of Mathematics, Massachusetts Institute of Technology, Cambridge, MA 02139 USA; 3 W. M. Keck Science Department, Pitzer, Scripps, and Claremont McKenna Colleges, Claremont, CA 91711 USA; 4 GlaxoSmithKline AI/ML, 200 Cambridgepark Drive, Cambridge, MA 02140 USA; 5 ITAMP, Harvard-Smithsonian Center for Astrophysics, Cambridge, MA 02138 USA; 6 Department of Physics, Harvard University, Cambridge, MA 02138 USA; 7 Research Laboratory of Electronics, Massachusetts Institute of Technology, Cambridge, MA 02139 USA; 8 Center for Theoretical Physics, Massachusetts Institute of Technology, Cambridge, MA 02139 USA; 9 Institute for Physical Science and Technology, University of Maryland, College Park, MD 20742 USA

**Keywords:** Statistical physics, Thermodynamics, Computational methods, Information storage, Magnetic materials

## Abstract

Diverse many-body systems, from soap bubbles to suspensions to polymers, learn and remember patterns in the drives that push them far from equilibrium. This learning may be leveraged for computation, memory, and engineering. Until now, many-body learning has been detected with thermodynamic properties, such as work absorption and strain. We progress beyond these macroscopic properties first defined for equilibrium contexts: We quantify statistical mechanical learning using representation learning, a machine-learning model in which information squeezes through a bottleneck. By calculating properties of the bottleneck, we measure four facets of many-body systems’ learning: classification ability, memory capacity, discrimination ability, and novelty detection. Numerical simulations of a classical spin glass illustrate our technique. This toolkit exposes self-organization that eludes detection by thermodynamic measures: Our toolkit more reliably and more precisely detects and quantifies learning by matter while providing a unifying framework for many-body learning.

## Introduction

Many-body systems can learn and remember patterns of drives that propel them far from equilibrium. Such behaviors have been predicted and observed in many settings, from charge-density waves^1,2^ to non-Brownian suspensions^3–5^, polymer networks^6^, soap-bubble rafts^7^, and macromolecules^8^. Such learning holds promise for engineering materials capable of memory and computation. Detecting such learning can also help us understand granular systems, e.g., infer the history of forces experienced by an asteroid core. This potential for applications, with experimental accessibility and ubiquity, have earned these classical nonequilibrium many-body systems much attention recently^9^.

A classical, randomly interacting spin glass exemplifies driven matter that learns. Let us call a set $\{ {\vec {A}}, {\vec {B}}, {\vec {C}} \}$ of magnetic fields a *drive*. Consider randomly selecting a field from the drive and applying it to the spin glass, then repeating this process many times. The spins absorb work from the fields. The power absorbed shrinks adaptively, in a certain parameter regime: The spins migrate toward a corner of configuration space where their configuration approximately withstands the drive’s insults. If new fields are imposed, the absorbed power spikes. If fields from $\{ {\vec {A}}, {\vec {B}}, {\vec {C}} \}$ are reimposed, the absorbed power spikes again, but less than under the unfamiliar fields^10^. The spin glass recognizes the original drive.

A simple, low-dimensional property of the material—absorbed power—distinguishes drive inputs that fit a pattern from drive inputs that do not. This property reflects a structural change in the spin glass’s configuration. The change is long-lived and not easily erased by new stimuli. For these reasons, we say that the material has *learned* the drive.

Many-body learning has been quantified with properties commonplace in thermodynamics. Examples include power, as explained above, and strain in polymers that learn stress amplitudes. Such thermodynamic diagnoses offer insights but suffer from two shortcomings. First, the thermodynamic properties vary from system to system. For example, work absorption characterizes the spin glass’s learning; strain characterizes non-Brownian suspensions’. A more general approach would facilitate comparisons and standardize analyses. Second, thermodynamic properties were defined for macroscopic equilibrium states. Such properties do not necessarily describe far-from-equilibrium systems’ learning optimally.

**Figure 1:**
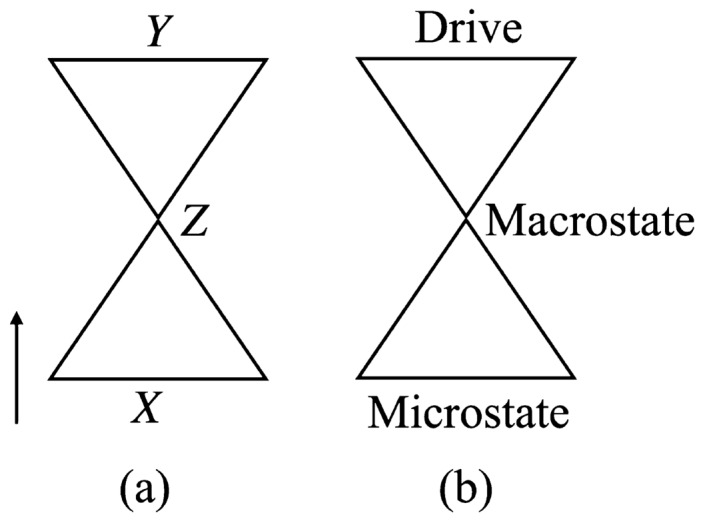
Parallel between two structures: (**a**) Bottleneck neural network, which performs representation learning. (**b**) Nonequilibrium-statistical-mechanics problem.

Separately from many-body systems’ learning, machine learning has flourished over the past decade^11,12^. Machine learning has helped elucidate how natural and artificial systems learn. Neural networks developed over the past decade can undergo *representation learning*^13^ (Fig. 1a). Such a neural network receives a high-dimensional variable *X*. Examples include a sentence missing a word, e.g., “The $\underline{\quad \quad }$ is shining.” The neural network compresses the input into a low-dimensional *latent variable* *Z*, e.g., word types and relationships. The neural network decompresses *Z* into a prediction ${\hat{Y}}$ of a high-dimensional variable *Y*. In the example, *Y* can be the word missing from the sentence, and ${\hat{Y}}$ can be “sun.” The size of the bottleneck *Z* controls a tradeoff between the memory consumed and the prediction’s accuracy. We call the neural networks that perform representation learning *bottleneck neural networks*.

**Figure 2:**
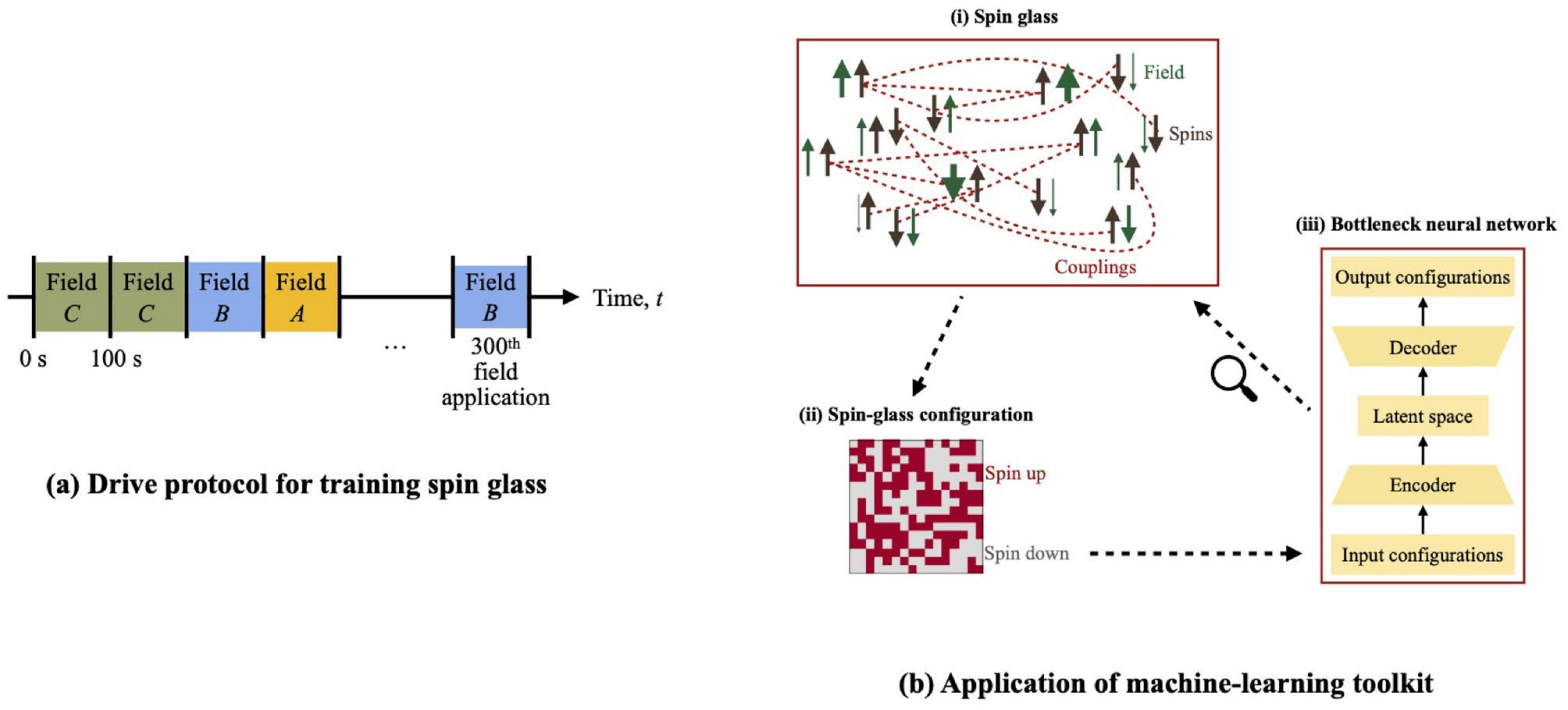
Schematic: (**a**) illustrates the protocol used to train the many-body system on a drive $\{A, B, C\}$. (**b**) sketches how the many-body system is driven into configurations on which the neural network trains. Analyzing the neural network’s latent space elucidates how much the many-body system has learned about its drive.

In this paper, we construct and deploy a bottleneck neural network to quantify how much many-body systems learn about the patterns of drives that force them: We use representation learning to learn how much many-body systems learn. Our measurement protocols share the following structure (Fig. 2b): The many-body system is trained with a drive (e.g., fields $\vec {A}$, $\vec {B}$, and $\vec {C}$). Then, the system is tested (e.g., with a field $\vec {D}$). Training and testing are repeated in many trials. Configurations realized by the many-body system are used to train a bottleneck neural network via unsupervised learning. Finally, we calculate properties of the neural network’s bottleneck. We illustrate with numerical simulations of the spin glass, whose learning has been detected with work absorption^10^. Our methods generalize to other platforms, however. This machine-learning toolkit offers three advantages.

1. Bottleneck neural networks register learning behaviors more thoroughly and precisely than work absorption.
2. Our framework encompasses a wide class of strongly driven many-body systems. Although we illustrate with the example of a spin glass, the framework does not rely on any particular thermodynamic property tailored to spins. Our neural network scores a many-body system’s learning behaviors with dimensionless numbers that can be compared across platforms.
3. Our approach unites machine learning with learning by many-body systems. The union is conceptually satisfying.

We measure four facets of many-body learning: classification ability, memory capacity, discrimination ability, and novelty detection. Our techniques, however, can be extended to other facets.

## Results

First, we introduce our bottleneck neural network. Then, we define the spin glass on which we will test our machine-learning toolkit. We finally show how to quantify, using representation learning, how much a many-body system learns about a drive.

### Bottleneck neural network

Representation learning, we argue, shares its structure with a problem in nonequilibrium statistical mechanics (Fig. 1b). Consider a many-body system subject to a strong drive. The system’s microstate occupies a high-dimensional space, like the input *X* to a bottleneck neural network. A macrostate synopsizes the microstate in a few numbers, such as particle number and magnetization. This synopsis parallels the latent space *Z*. If the many-body system has learned the drive, the macrostate encodes the drive. One may reconstruct the drive from the macrostate, as a bottleneck neural network reconstructs *Y* from *Z*. See Ref.^14^ for a formal parallel between representation learning and equilibrium thermodynamics.

We construct a neural network inspired by this parallel. As the macrostate informs computations in the statistical-mechanics problem described above, the neural network’s bottleneck informs our computations. One might initially aim for a bottleneck neural network that predicts drives from configurations *X*. But such a neural network would undergo supervised learning, if constructed according to the state of the art of when this paper was written. During supervised learning, the neural network receives tuples (configuration of the many-body system, label of drive that generated the configuration). The drive labels are not directly available to the many-body system. So successful predictions by neural network predictions would not necessarily reflect only learning by the many-body system. Hence we design a bottleneck neural network that performs unsupervised learning, receiving only configurations.

This neural network is a *variational autoencoder*^15–17^, a generative model: It receives samples *x* from a distribution over the possible *X* values, creates a variational model for the distribution, and samples from the model. The model is refined via Bayesian variational inference (see Supplementary Note I for an overview). The model’s parameters are optimized via backpropagation during training.

Our variational autoencoder has five fully connected hidden layers, with neuron numbers 200-200-(number of *Z* neurons)-200-200. We usually restrict the latent variable *Z* to 2–4 neurons. This choice enables us to visualize the latent space and suffices to quantify the spin glass’s learning. Growing the many-body system may require more latent dimensions, as may growing the number of drives whose patterns the many-body system must learn. But our studies suggest that the number of dimensions needed $\ll$ the system size.

**Figure 3:**
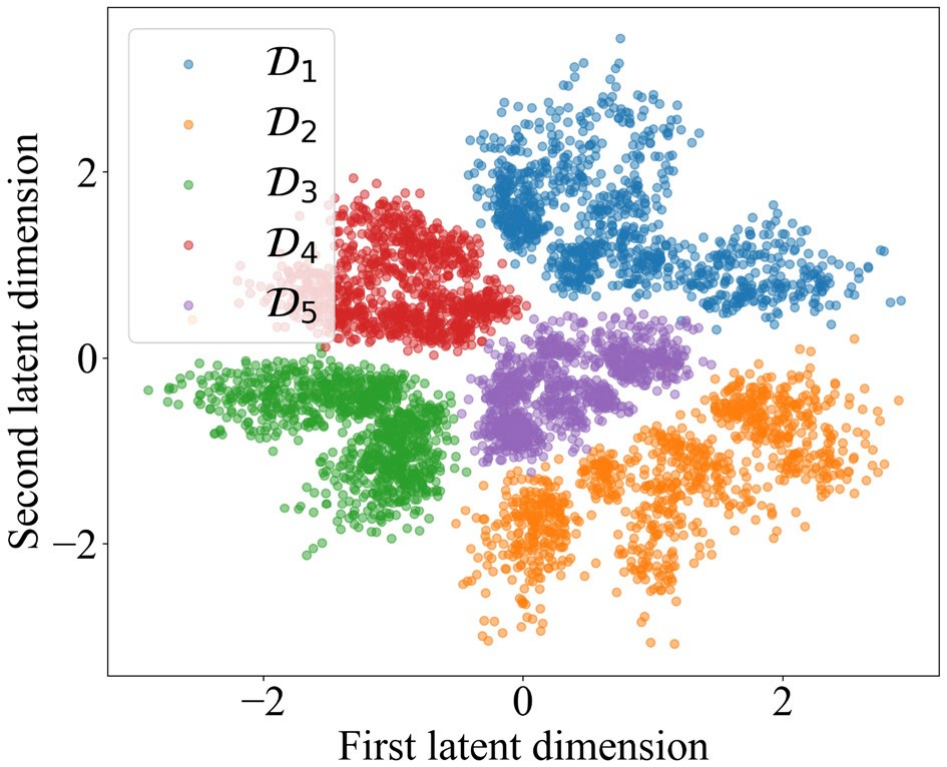
Visualization of latent space, *Z*: *Z* consists of neurons $Z_1$, represented along the *x*-axis, and $Z_2$, represented along the *y*-axis. A variational autoencoder formed *Z* while training on configurations assumed by a 256-spin glass exposed to different drives in different trials. The neural network mapped each configuration to a dot in latent space. After the training completed, each dot was colored according to which drive produced the corresponding configuration.

Figure 3 depicts the latent space *Z*. Each neuron corresponds to one axis and represents a continuous-valued real number. The latent space was formed via the protocol detailed below, in the section “How to quantify a many-body system’s learning of a drive, using representation learning.” To synopsize, we trained the spin glass on one drive in each of 1000 trials; trained the spin glass in another drive in each of 1000 trials; and so on, for five drives total. On the end-of-trial spin-glass configurations, the neural network was trained. The neural network compressed each configuration to a dot in latent space. We colored each dot according to which drive produced the corresponding configuration. We added the colors after the neural network’s training, so the neural network received no configurations’ drive labels. Same-color dots cluster together, so the spin glass distinguished the drives, as recognized by the neural network.

One might wonder whether our toolkit requires deep learning. Could simpler algorithms detect and measure many-body learning as sensitively? Supplementary Note II responds negatively. We compare our neural network with simpler competitors that perform unsupervised learning: a single-layer linear neural network, related to principal-component analysis^18^, and a clustering algorithm. The bottleneck neural network outperforms both competitors. (Competitors that perform supervised learning would enjoy an unfair advantage and, as explained above, would not reflect the many-body’s system learning faithfully.)

### Spin glass

A spin glass exemplifies the many-body learner^10^. We illustrate our machine-learning toolkit by simulating a glass of $N= 256$ classical spins. The *j*th spin occupies one of two possible states: $s_j = \pm 1$.

The spins couple together and experience an external magnetic field: Spin *j* evolves under a Hamiltonian

1 $$\begin{aligned} H_j(t) = \sum _{k \ne j} J_{jk} s_j s_k + A_j(t) s_j , \end{aligned}$$

and the spin glass evolves under $H(t) = {\frac{1}{2}} \sum _{j = 1}^NH_j(t)$, at time *t*. We call the first term in Eq. (1) the *interaction energy* and the second term the *field energy*. The couplings $J_{j k} = J_{kj}$ are defined through an Erdös-Rényi random network: Spins *j* and *k* have some probability *p* of interacting, for all *j* and $k \ne j$. Each spin couples to eight other spins, on average. The nonzero couplings $J_{j k}$ are selected according to a normal distribution of standard deviation 1.

$A_j(t)$ denotes the magnitude and sign of the external field experienced by spin *j* at time *t*. The field always points along the same direction (the *z*-axis), so we omit the arrow from $\vec {A}_j(t)$. We will simplify the notation for the field from $\{ A_j(t) \}_j$ to *A* (or *B*, etc.). Each $A_j(t)$ is selected according to a normal distribution of standard deviation 3. The field changes every 100 s.

To train the spin glass, we construct a drive by constructing a set $\{A, B, \ldots \}$ of random fields. We randomly select a field from the set, then apply the field for 100 s. This selection-and-application process is performed 300 times (Fig. 2a).

The spin glass exchanges heat with a bath at a temperature $T = 1 / \beta$. We set Boltzmann’s constant to $k_\text{B}= 1$. Energies are measured in Kelvins (K). To flip, a spin must overcome a height-*B* energy barrier. Spin *j* tends to flip at a rate $\omega _j = e^{\beta [ H_j(t) - B]} / (1 \text { s}).$ This rate has the form of Arrhenius’s law and obeys detailed balance. The average spin flips once per $10^7$ s. We model the evolution with discrete 100-s time intervals, using the Gillespie algorithm.

The spins absorb work when the field changes, as from $\{ A_j(t) \}$ to $\{ A'_j(t) \}$. The change in the spin glass’s energy equals the work absorbed by the spin glass: $W := \sum _{j = 1}^N\left[ A'_j(t) - A_j(t) \right] s_j.$ Absorbed power is defined as $W / ( \text {100~s} )$. The spin glass dissipates heat by losing energy as spins flip.

The spin glass is initialized in a uniformly random configuration ${\mathcal {C}}$. Then, the spins relax in the absence of any field for 100,000 s. The spin glass navigates to near a local energy minimum. If a protocol is repeated in multiple trials, all the trials begin with the same configuration ${\mathcal {C}}$.

In a certain parameter regime, the spin glass learns its drive effectively, even according to the absorbed power^10^. Consider training the spin glass on a drive $\{ A, B, C \}$. The spin glass absorbs much work initially. If the spin glass learns the drive, the absorbed power declines. If a dissimilar field *D* is then applied, the absorbed power spikes. If the familiar fields are reapplied, the absorbed power spikes again, but less. The spin glass learns effectively in the “Goldilocks regime” of $\beta = 3$ K$^{-1}$ and $B = 4.5$ K^10^: The temperature is high enough, and the barriers are low enough, that the spin glass can explore phase space. But *T* is low enough, and the barriers are high enough, that the spin glass is not hopelessly peripatetic.

Spins can fail to learn nontrivially, yet adopt configurations that reflect a drive. For example, the spins can be entrained to the field. The spins would bear the field’s stamp as silly putty bears a thumbprint. A thumbprint vanishes as soon as the silly putty is smoothed. Hence the silly putty undergoes no long-lived structural change that resists erasure; the silly putty does not learn robustly. Alternatively, most of the spins can remain frozen, while only a few flip. One might infer the drive from the few flippable spins, though most of the glass would contain no information about the drive. We confirm that our spin glass does not exhibit these behaviors, in Supplementary Note III: the spin glass’s learning is nontrivial.

### How to quantify a many-body system’s learning of a drive, using representation learning

We detect and quantify four facets of learning: classification ability, memory capacity, discrimination, and novelty detection. One *classifies* a stimulus by answering the question “Which of the possible stimuli is this one?” A system’s *memory capacity* is the number of fields that the system can remember. (We use the term “memory capacity” in the physical sense of Ref.^9^. A more specific, technical definition of “memory capacity” is used in reservoir computing^19^.) One performs *novelty detection* by answering the question “Have I encountered this stimulus before?” One *discriminates* between stimuli *A* and *B* by answering “How much of the present stimulus consists of *A*, and how much consists of *B*?”

Below, we illustrate the application of our toolkit by quantifying classification ability. The Methods show how to apply our toolkit to the other three facets of learning. Further facets may be quantified similarly. Our machine-learning approach detects and measures learning more reliably and precisely than absorbed power does. Code used and data generated are accessible at Ref.^20^.

A system *classifies* a stimulus when identifying the stimulus as one of many possibilities. First, we detail the protocol run on the spin glass. Second, we show how to measure the spin glass’s classification ability using representation learning. Third, we measure the spin glass’s classification ability using absorbed power. The neural network, we find, reflects more of the spin glass’s classification ability than absorbed power does.

The spin glass underwent the following protocol. We generated random fields *A*, *B*, *C*, *D*, and *E*. From 4 of the fields, we formed the drive ${\mathcal {D}}_1 := \{A, B, C, D\}$. On the drive, we trained the spin glass in each of 1000 trials. In each of 1000 other trials, we trained a refreshed spin glass on a drive ${\mathcal {D}}_2 := \{A, B, C, E\}$. We repeated this process for each of the 5 possible 4-field drives. Ninety percent of the trials were randomly selected for training the neural network. The rest were used for testing.

We measured the spin glass’s ability to classify drives, using the neural network, as follows: We fixed a time *t*, then identified the configurations occupied by the spin glass at *t* in the spin-glass-training trials. On these configurations, we trained the neural network. The neural network populated the latent space with dots (similarly to in Fig. 3). The dots generated by drive ${\mathcal {D}}_j$ approximated a probability density $P_j$, for all $j = 1, 2, 3, 4, 5$.

**Figure 4:**
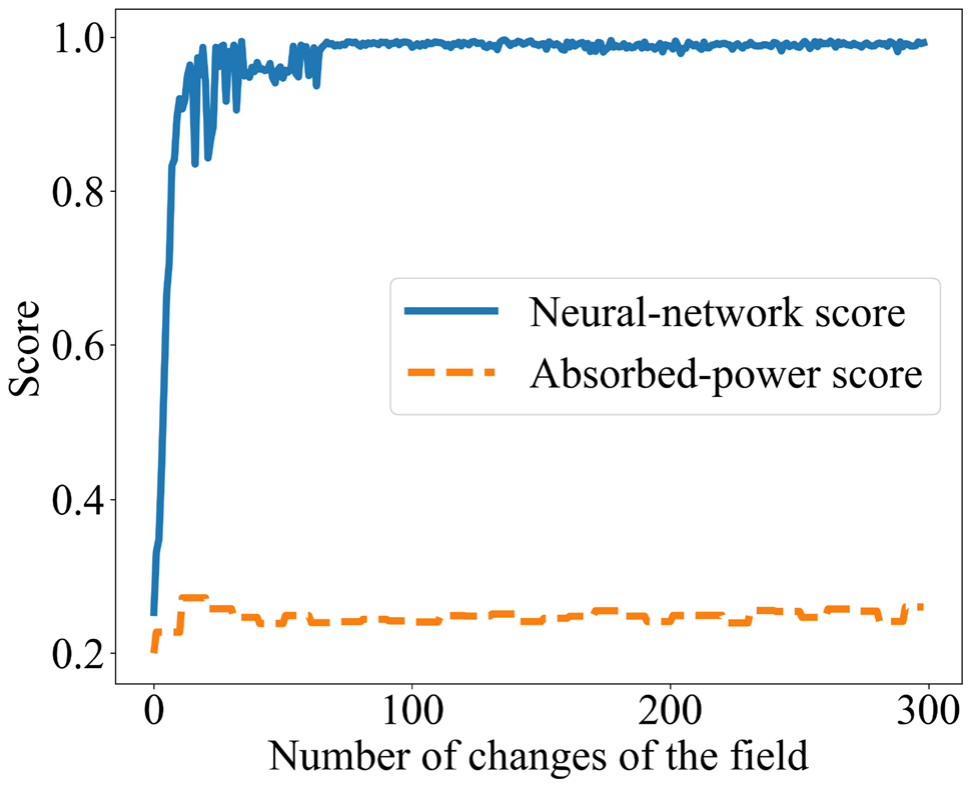
Quantification of a many-body system’s classification ability: A spin glass classified a drive as one of five possibilities. The blue, upper curve represents the system’s classification ability, as quantified by a bottleneck neural network. The orange, lower curve represents the classification ability as quantified with the absorbed power. The neural-network score rises to near the maximum, 1.00. The thermodynamic score exceeds the random-guessing score, 1/5, slightly. The neural network therefore detects more of the spins’ classification ability.

We then gave the neural network a time-*t* configuration from a test trial. The neural network compressed the configuration into a latent-space point. We calculated the probability that drive ${\mathcal {D}}_j$ generated that point, using $P_j$, for all *j*. The highest-probability drive most likely generated the point, by maximum-likelihood estimation^21^. We performed this testing and estimation for each trial in the test data. The fraction of trials in which the estimation succeeded constitutes the *score*. The score is plotted against *t* in Fig. 4 (blue, upper curve).

We compare with the classification ability attributed to the spin glass by the absorbed power: We fixed a drive ${\mathcal {D}}_j$ and a time *t*. We identified the neural-network-training trials in which ${\mathcal {D}}_j$ was applied at time *t*. From the power absorbed then, we formed a histogram. We performed this process for each drive ${\mathcal {D}}_j$. Then, we took a trial from the test set and identified the power absorbed at *t*. We inferred which drive most likely produced that power, applying maximum-likelihood estimation to the histograms. The guess’s score appears as the orange, lower curve in Fig. 4.

A score maximizes at 1.00 if the drive is always guessed accurately. The score is lower-bounded by the random-guessing value $1 / (\text {number of drives}) = 1/5$. In Fig. 4, each score grows over tens of field switches. The absorbed-power score begins at 0.20 and comes to fluctuate around 0.25. The neural network’s score comes to fluctuate slightly below 1.00. (The neural network’s score begins slightly above 0.20. One might expect the score to begin at 0.20: At $t = 0$, the spin glass has not experienced the drive, so the neural network receives no information about the drive, so the neural network can guess the drive only randomly. The distance from 0.20, we expect, comes from stochasticity of three types: the spin glass’s initial configuration, maximum-likelihood estimation, and stochastic gradient descent. Stochasticity of only the first two types affects the absorbed-power score.) Hence the neural network detects more of the spin glass’s classification ability than the absorbed power does, in addition to suggesting a means of quantifying the classification ability rigorously. Having illustrated our machine-learning toolkit with classification, we detail applications to memory capacity, novelty detection, and discrimination in the Methods.

## Discussion

We have detected and quantified a many-body system’s learning of drive patterns, using representation learning. Our toolkit affords greater sensitivity than absorbed power, a representative of the thermodynamic toolkit applied to detect many-body learning until now. Our technique quantifies classification ability, memory capacity, discrimination ability, and novelty detection. The toolkit is general, not relying on whether the system exhibits magnetization or strain or another thermodynamic response. The Methods establish the feasibility of applying our toolkit in a variety of experiments and simulations. This approach provides a framework for understanding memory—a basic, widely realized, and usable trait—in a unified manner across classical statistical mechanics. This framework opens several opportunities for future research; we detail two below.

First, our toolkit is well-suited to more open problems about many-body learners. An example problem concerns the soap-bubble raft in Ref. ^7^. Experimentalists trained a raft of soap bubbles with an amplitude-$\gamma _\text{t}$ strain. The soap bubbles’ positions were tracked, and variances in positions were calculated. No such measures distinguished trained rafts from untrained rafts; only stressing the raft and reading out the strain could^7,22^. Our bottleneck neural network is well-poised to identify microscopic properties that distinguish trained from untrained rafts. Similarly, representation learning may facilitate the detection of active matter. Self-organization is detected now through simple, large-scale, easily visible signals^23^. Bottleneck NNs could identify patterns invisible in thermodynamic measures.

Second, in statistical mechanics, we parameterize macrostates with volume, energy, magnetization, and other thermodynamic variables. Macrostates in statistical mechanics parallel the latent space in our bottleneck neural network (Fig. 1). Which variables parameterize the neural network’s latent space? Latent space may suggest definitions of new thermodynamic variables, or hidden relationships amongst known thermodynamic variables.

**Figure 5:**
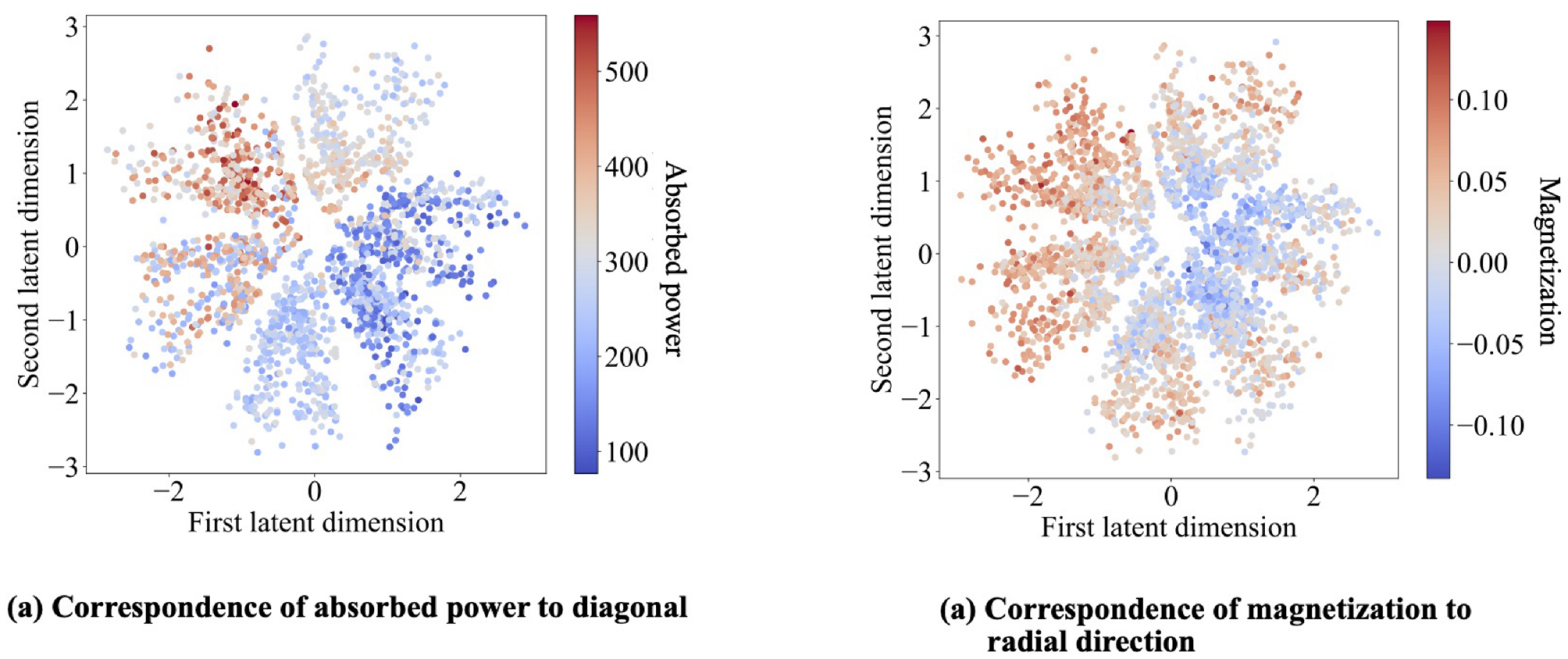
Correspondence of latent-space directions to thermodynamic quantities: A variational autoencoder trained on the configurations assumed by a spin glass exposed to fields *A*, *B*, and *C*. We have color-coded each latent-space plot, highlighting how a thermodynamic property changes along some direction. In (**a**), the absorbed power grows from the bottom righthand corner to the upper lefthand corner. In (**b**), the magnetization grows radially.

We illustrate by training the spin glass with a drive $\{ A, B, C \}$ in each of many trials. On the end-of-trial configurations, we trained the neural network. Two latent-space directions have physical significances, as shown in Fig. 5: the absorbed power grows along the diagonal from the bottom righthand corner to the upper lefthand corner (Fig. 5a). The magnetization grows radially (Fig. 5b). The directions are nonorthogonal, suggesting a nonlinear relationship between the thermodynamic variables. Convention biases physicists toward measuring volume, magnetization, heat, work, etc. The neural network may identify new macroscopic variables better-suited to far-from-equilibrium statistical mechanics, or nonlinear relationships amongst thermodynamic variables.

We can translate, as follows, between conventional thermodynamic variables and the latent-space directions $z_1$ and $z_2$: List the conventional thermodynamic variables expected to be relevant: $v_1, v_2, \ldots , v_n$. For example, $v_1$ may denote the work absorption, and $v_2$ may denote the magnetization. The neural network populates the latent space with dots during training. Each dot corresponds to $v_j$’s calculable from the corresponding many-body configuration. A feedforward neural network can decompose each $z_k$ as a function of the $v_j$’s. We will have decomposed the latent-space variables in terms of thermodynamic variables, translating between the two. A bottleneck neural network could uncover new theoretical physics, as discussed in, e.g., Refs.^24–26^.

## Methods

In the Results, we applied our machine-learning toolkit to quantify classification ability. Here, we apply the toolkit to quantify three more facets of learning: memory capacity, discrimination, and novelty detection. We also demonstrate the feasibility of applying our toolkit to experiments.

### Memory capacity: How many fields can the system remember?

How many fields can a many-body system remember? A bottleneck neural network, we find, registers a greater memory capacity than absorbed power registers. Hence the neural network reflects statistical mechanical learning, at high field numbers, that the absorbed power does not.

We illustrate by constructing 50 random fields. We selected 40 to form a drive ${\mathcal {D}}_1$, selected 40 to form a drive ${\mathcal {D}}_2$, and repeated until forming five drives. We trained the spin glass on drive ${\mathcal {D}}_j$ in each of 1000 trials, for each of $j = 1, 2, \ldots 5$. Ninety percent of the trials were designated as neural-network-training trials; and 10%, as neural-network-testing trials.

The choice of 50 fields is explained in Supplementary Note IV: 50 fields exceed the spin-glass capacity registered by the absorbed power. We will show that 50 fields do not exceed the capacity registered by the neural network: The neural network identifies spin-glass learning missed by the absorbed power.

**Figure 6:**
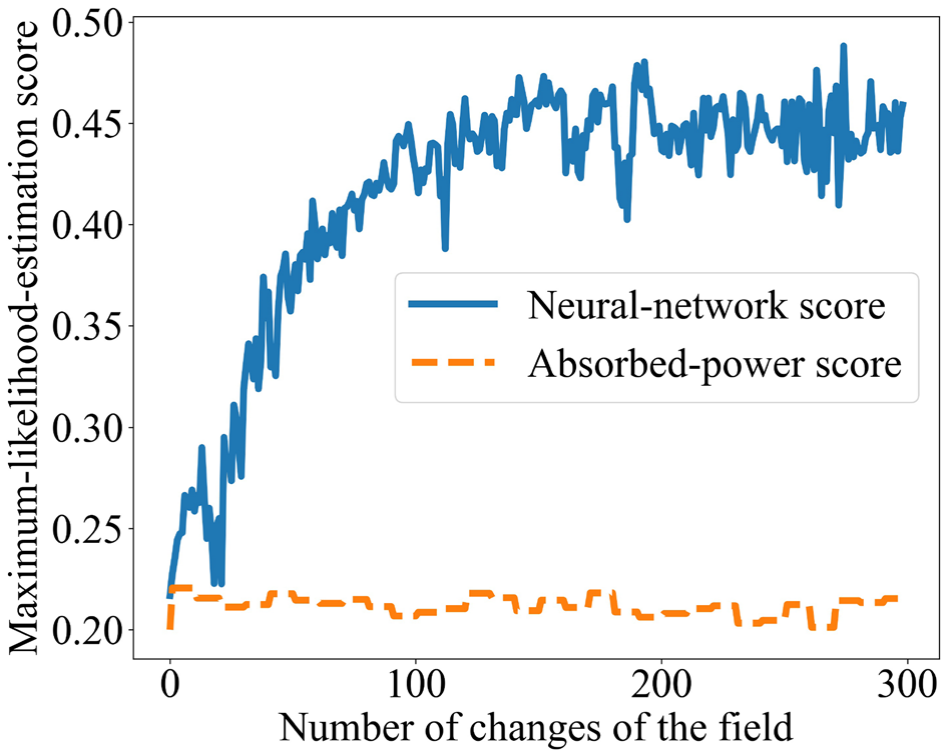
Quantification of memory capacity: A spin glass was trained on one of five drives in each of many trials. Each drive was formed from 40 fields selected from 50 random fields. The upper, blue line represents the memory capacity attributed to the spin glass by a bottleneck neural network. The lower, orange line represents the memory capacity attributed by absorbed power.

We used representation learning to quantify the spin glass’s capacity as follows. For a fixed time *t*, we collected the configurations occupied by the spin glass at *t* in the neural-network-training trials. On these configurations, the neural network performed unsupervised learning. The neural network populated its latent space with dots that formed five clusters. The cluster sourced by drive ${\mathcal {D}}_j$ approximated a probability density $P_j$. We fed the neural network the configuration occupied at *t* during a test trial. The neural network formed a new dot in latent space. We estimated the probability that drive ${\mathcal {D}}_j$ formed the drive, using $P_j$, for each *j*. The greatest probability stemmed from the drive ${\mathcal {D}}_j$ that most likely, according to the neural network, produced the point. That is, we applied maximum-likelihood estimation. The fraction of test trials in which the neural network guessed correctly constitutes the neural network’s score. The score is plotted against *t* in Fig. 6, as the blue, upper curve.

The neural network’s score is compared with the absorbed power’s score, calculated as follows. For a fixed time *t*, we identified the power absorbed at *t* in each neural-network-testing trial. We histogrammed the power absorbed when ${\mathcal {D}}_j$ was applied at *t*, for each $j = 1, 2, \ldots , 5$. We then identified the power absorbed at *t* in a test trial. Comparing with the histograms, we inferred which drive was most likely being applied. We repeated this inference with each other test trial. In which fraction of the trials did the absorbed power identify the drive correctly? This number forms the absorbed power’s score. The score is plotted as the lower, orange curve in Fig. 6.

The higher the score, the greater the memory capacity attributed to the spin glass. The absorbed power identifies the drive in approximately $20\%$ of the trials, as would random guessing. The score remains approximately constant, because the number of fields exceeds the spin-glass capacity measured by the absorbed power. In contrast, the neural network’s score grows over $\approx 150$ changes of the field, then plateaus at $\approx 0.450$. The neural network points to the wrong drive most of the time but succeeds significantly more often than the absorbed power. Hence representation learning uncovers more of the spin glass’s memory capacity than absorbed power measure does.

In summary, a many-body system’s memory capacity can be quantified as the greatest number of fields in any drive on which maximum-likelihood estimation, based on a neural network’s latent space, scores better than random guessing.

### Discrimination: How new is this field?

Suppose that a many-body system learns fields *A* and *B*, then encounters a field that interpolates between them. Can the system recognize that the new field contains familiar constituents? Can the system discern how much *A* contributes and how much *B* contributes? The answers characterize the system’s discrimination ability, which we quantify with a maximum-likelihood-estimation score. Estimates formed from the neural network’s latent space reflect more of the system’s discriminatory ability than do estimates formed from absorbed power.

We illustrate with the spin glass, forming a drive $\{A, B, C\}$. Each trial began with 300 subsequent time intervals. In each interval, a field was selected randomly from the drive and applied. The spin glass was then tested with a linear combination $D_w = w A + (1 - w) B$. The weight *w* varied from 0 to 1, in steps of 1/6, across trials.

We measured the spin glass’s discrimination using the neural network as follows. We identified the final configuration assumed by the spin glass in each trial. These configurations were split into neural-network-training data and neural-network-testing data. The training trials ended with configurations on which the neural network was trained. Then, the neural network received a configuration with which a neural-network-testing trial ended. The neural network mapped the configuration to a latent-space point. We inferred which field most likely generated that point, using maximum-likelihood estimation on the latent space. We tested the neural network with all the test trials. The fraction of maximum-likelihood estimates that were correct formed the neural network’s score.

Similarly, we measured the spin glass’s discrimination using the absorbed power. We fixed a value of *w*, then identified the neural-network-training trials that ended with the application of $D_w$. We identified the power ${\mathcal {P}}$ absorbed by the spin glass after the $D_w$ application. We histogrammed ${\mathcal {P}}$, inferring the probability that, if shown $D_w$ for a given *w*, the spin glass will absorb an amount ${\mathcal {P}}$ of power. We formed a histogram for each value of *w*. Then, we calculated the power absorbed during a neural-network-testing trial. We inferred which field most likely generated that point, applying maximum-likelihood estimation to the histograms. We repeated the maximum-likelihood estimation with each neural-network-testing trial. The absorbed power’s score equals the fraction of the trials in which the maximum-likelihood estimation was correct.

The neural network’s score equals about double the absorbed power’s score, for latent spaces of dimensionality 2–20. The neural network scores between 0.448 and 0.5009, whereas the absorbed power scores 0.2381. Hence the representation-learning model picks up on more of the spin glass’s discriminatory ability than the absorbed power does.

In summary, a many-body system’s ability to discriminate amongst combinations of familiar fields can be quantified with the score of maximum-likelihood estimates formed from a neural network’s latent space.

### Novelty detection: Has the system encountered this drive before?

At the start of the introduction, we described how absorbed power has been used to identify novelty detection. A system detects novelty when labeling a stimulus as familiar or unfamiliar. The stimulus produces a response that exceeds a threshold or lies below. If the stimulus exceeds the threshold, an observer should guess that the stimulus is novel. Otherwise, the observer should guess that the stimulus is familiar.

The observer can err in two ways: One commits a *false positive* by believing a familiar drive to be novel. One commits a *false negative* by believing a novel drive to be familiar. The errors trade off: Raising the threshold lowers the probability $p( {\text {pos.}}|{\text {neg.}})$, suppressing false positives at the cost of false negatives. Lowering the threshold lowers the probability $p({\text {neg.}}|{\text {pos.}})$, suppressing false negatives at the cost of false positives.

**Figure 7:**
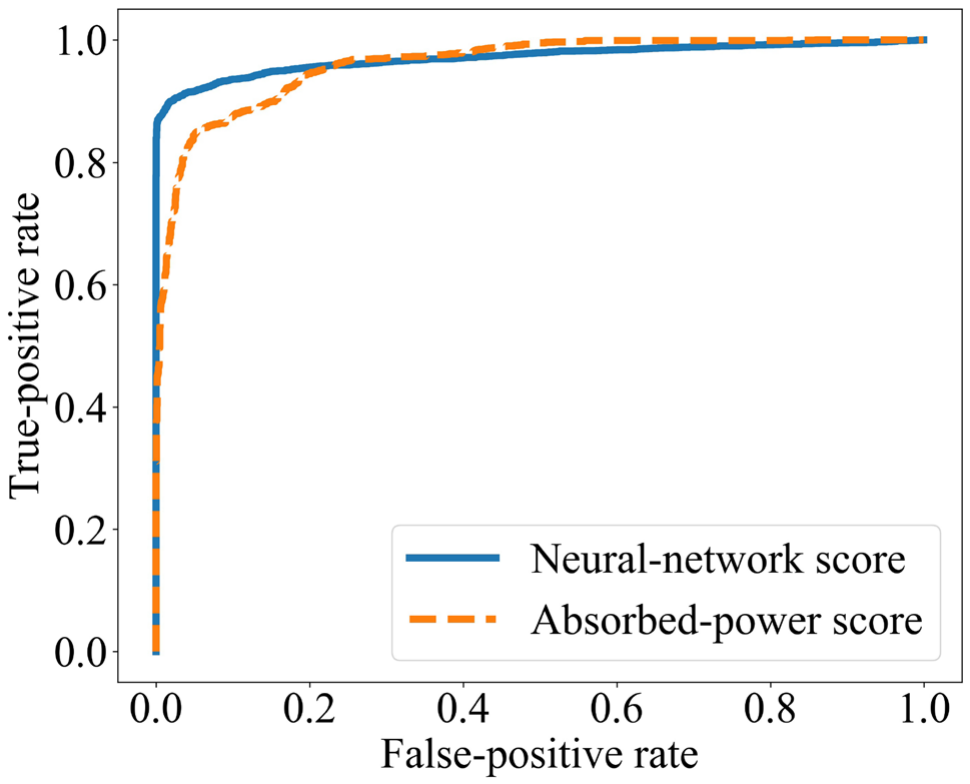
Receiver-operating-characteristic (ROC) curve: The spin glass was trained with three drives, then tested with a familiar drive or with a novel drive. From a response of the system’s, an ROC curve can be defined. The blue, solid curve is defined in terms of a bottleneck neural network; and the orange, dashed curve is defined in terms of absorbed power.

The *receiver-operating-characteristic* (ROC) curve depicts the tradeoff’s steepness (see Ref.^27^ and Fig. 7). Each point on the curve corresponds to one threshold value. The false-positive rate $p( {\text {pos.}}|{\text {neg.}} )$ runs along-the *x*-axis; and the true-positive rate, $p( {\text {pos.}}|{\text {pos.}})$, along the *y*-axis. The greater the area under the ROC curve, the more sensitively the response reflects accurate novelty detection.

We measure a many-body system’s novelty-detection ability using an ROC curve. Let us illustrate with the spin glass. We constructed two random drives, $\{A, B, C \}$ and $\{D, E, F \}$. We trained the spin glass on $\{A, B, C \}$. In each of 3,000 trials, we then tested the spin glass with *A*, *B*, or *C*. In each of 3000 other trials, we tested with *D*, *E*, or *F*. We defined one response in terms of a bottleneck neural network, as detailed below; measured the absorbed power; and, from each response, drew an ROC curve (Fig. 7). The curves show that representation learning and absorbed power detect the spin glass's novelty detection about equally well. Each method excels slightly in one regime or another.

We defined the representation-learning response as follows. We trained the neural network on the configurations assumed by the spin glass during its training. The neural network populated latent space with three clumps of dots. We modeled the clumps with a hard mixture $p_{ABC} (z_1, z_2)$ of three Gaussians. (A mixture is hard if it models each point as belonging to only one Gaussian.) We then fed the neural network the configuration that resulted from testing the spin glass. The neural network mapped the configuration to a latent-space point $(z_1^\text{test}, z_2^\text{test})$. We calculated the probability $p_{ABC} (z_1^\text{test}, z_2^\text{test}) \, dz_1 dz_2$ that the *ABC* distribution produced the new point. This probability was compared to a fixed threshold. If the probability exceeded the threshold, the test configuration was guessed to have been produced by a novel drive. We repeated this protocol with the other test trials, using the fixed threshold. The fraction of guesses that were true positives, and the fraction of guesses that were false positives, specified one point on the blue, solid curve in Fig. 7. Varying the threshold led to the other points.

We defined a thermodynamic ROC curve in terms of absorbed power. Consider the trials in which the spin glass is tested with field *A*. We histogrammed the power absorbed by the spin glass after the *A* test. We formed another histogram from the *B*-test trials; and a third histogram, from the *C*-test trials. To these histograms was compared the power ${\mathcal {P}}$ that the spin glass absorbed during a test with an arbitrary field. We inferred the likelihood that ${\mathcal {P}}$ resulted from a familiar field. The results form the orange, dashed curve in Fig. 7.

The two ROC curves enclose regions of approximately the same area: the neural network curve encloses an area-0.9633 region; and the thermodynamic curve, an area-0.9601 region. On average across all thresholds, therefore, the responses register novelty detection approximately equally. Yet the responses excel in different regimes: The neural network achieves greater true-positive rates at low false-positive rates, and the absorbed power achieves greater true-positive rates at high false-positive rates. This two-regime behavior persisted across batches of trials, though the enclosed areas fluctuated slightly. Hence anyone paranoid about avoiding false positives should measure a many-body system’s novelty detection with a neural network. Those more relaxed might prefer the absorbed power.

Why should the neural network excel at low false-positive rates? Because of the neural network’s skill at generalizing, we expect. Upon training on cat pictures, a neural network generalizes from the instances. Shown a new cat, the neural network recognizes its catness. Perturbing the input a little perturbs the neural network’s response little. Hence changing the magnetic field a little, which changes the spin-glass configuration little, should change latent space little, obscuring the many-body system’s novelty detection. This obscuring disappears when the magnetic field changes substantially.

In summary, a many-body system’s novelty-detection ability is quantified with an ROC curve formed from a neural network’s latent space or a thermodynamic response, depending on the false-positive threshold.

### Feasibility

Applying our toolkit might appear impractical, since microstates must be inputted into the neural network. Measuring a many-body system’s microstate may daunt experimentalists. Yet the use of microstates hinders our proposal little, for three reasons.

First, microstates can be calculated in numerical simulations, which inform experiments. Second, many key properties of many-body microstates have been measured experimentally. High-speed imaging has been used to monitor soap bubbles’ positions^7^ and colloidal suspensions^28^. Similarly wielded tools, such as high magnification, have advanced active-matter^29^ and gene-expression^30^ studies.

One might worry that the full microstate cannot be measured accurately or precisely. Soap bubbles’ positions can be measured with finite precision, and other microscopic properties might be inaccessible. But, third, some bottleneck neural networks denoise their inputs^12,31^: The neural networks learn the distribution from which samples are generated ideally, not systematic errors. Denoising by variational autoencoders is less established but is progressing^32^.

## Supplementary information

Supplementary Information.

## Data Availability

The machine-learning and spin-glass-simulation code is available at Ref.^20^. Will be available at^20^ once COVID-19 restrictions loosen enough that we can access the computers that store the files.
