# Supplementary material for: Machine learning outperforms thermodynamics in measuring how well a many-body system learns a drive
Source: Sci Rep. 2021 Apr 29;11:9333. doi: 10.1038/s41598-021-88311-7 (PMC8085166; doi:10.1038/s41598-021-88311-7)
Supplement: Supplementary file 1 — Supplementary Information. [file 41598_2021_88311_MOESM1_ESM.pdf]

Supplementary Information for “Machine learning outperforms thermodynamics in measuring how well a many-body system learns a drive”

Weishun Zhong, Jacob M. Gold, Sarah Marzen, Jeremy L. England, and Nicole Yunger Halpern

ABSTRACT

Supplementary Note I details the bottleneck neural network used to measure the many-body system's learning abilities. Supplementary Note II justifies our use of machine learning: We show that the bottleneck neural network outperforms competitors in measuring the many-body system's learning abilities. Supplementary Note III distinguishes the spin glass's learning from two trivial, superficially similar behaviors. Supplementary Note IV confirms that 50 fields exceed the memory capacity attributed to the spin glass by the absorbed power.

I Details about the bottleneck neural network

We briefly motivate and review variational autoencoders, then describe the variational autoencoder applied in the main text. Further background about variational autoencoders can be found in[1–3]. We denote vectors with boldface in this appendix.

Denote by $\mathbf{X}$ data that has a probability $p_{\boldsymbol{\theta}}(\mathbf{x})$ of assuming the value $\mathbf{x}$. $\boldsymbol{\theta}$ denotes a parameter, and $p_{\boldsymbol{\theta}}(\mathbf{x})$ is called the *evidence*. We do not know the form of $p_{\boldsymbol{\theta}}(\mathbf{x})$, when using representation learning. We model $p_{\boldsymbol{\theta}}(\mathbf{x})$ by identifying latent variables $\mathbf{Z}$ that assume the possible values $\mathbf{z}$. Let $p_{\boldsymbol{\theta}}(\mathbf{x}|\mathbf{z})$ denote the conditional probability that $\mathbf{X}=\mathbf{x}$, given that $\mathbf{Z}=\mathbf{z}$. We model the evidence, using the latent variables, with

$$p_{\boldsymbol{\theta}}(\mathbf{x}) = \int d\mathbf{z}\, p_{\boldsymbol{\theta}}(\mathbf{x}|\mathbf{z}) p(\mathbf{z}). \tag{1}$$

$p_{\boldsymbol{\theta}}(\mathbf{x}|\mathbf{z})$ can be related to the posterior distribution $p_{\boldsymbol{\theta}}(\mathbf{z}|\mathbf{x})$. The posterior is the probability that, if $\mathbf{X}=\mathbf{x}$, then $\mathbf{Z}=\mathbf{z}$. By Bayes' rule, $p_{\boldsymbol{\theta}}(\mathbf{z}|\mathbf{x}) = p_{\boldsymbol{\theta}}(\mathbf{x}|\mathbf{z})p(\mathbf{z})/p_{\boldsymbol{\theta}}(\mathbf{x})$. Calculating the posterior is usually impractical, as $p_{\boldsymbol{\theta}}(\mathbf{x})$ is typically intractable (cannot be calculated analytically). Hence we approximate the posterior with a variational model $q_{\boldsymbol{\phi}}(\mathbf{z}|\mathbf{x})$. The optimization parameter $\boldsymbol{\phi}$ denotes the neural network's weights and biases.

The approximation introduces an inference error, quantified with the Kullback-Leibler divergence. Let $P(\mathbf{u})$ and $Q(\mathbf{u})$ denote distributions over the possible values $\mathbf{u}$ of a variable. The Kullback-Leibler divergence quantifies the distance between the distributions:

$$D_{\text{KL}}(P(\mathbf{u})||Q(\mathbf{u})) := \mathbb{E}_{P(\mathbf{u})}[\ln P(\mathbf{u})] - \mathbb{E}_{P(\mathbf{u})}[\ln Q(\mathbf{u})] \tag{2}$$

$$\geq 0. \tag{3}$$

$\mathbb{E}_{P(\mathbf{u})}[f(\mathbf{u})]$ denotes the average of a function $f(\mathbf{u})$ over the distribution $P(\mathbf{u})$. Operationally, the Kullback-Leibler divergence equals the maximal efficiency with which the distributions can be distinguished, on average, in a binary hypothesis test. We quantify our inference error with the Kullback-Leibler divergence between the variational model and the posterior, $D_{\text{KL}}(q_{\boldsymbol{\phi}}(\mathbf{z}|\mathbf{x})||p_{\boldsymbol{\theta}}(\mathbf{z}|\mathbf{x}))$.

Recall that we wish to estimate $p_{\boldsymbol{\theta}}(\mathbf{x})$: An accurate estimate lets us predict $\mathbf{x}$ accurately. We wish also to estimate the latent posterior distribution, $q_{\boldsymbol{\phi}}(\mathbf{z}|\mathbf{x})$. We therefore write out the Kullback-Leibler divergence's form, apply Bayes' rule to rewrite the $p_{\boldsymbol{\theta}}(\mathbf{z}|\mathbf{x})$, rearrange terms, and repackage terms into a new Kullback-Leibler divergence:

$$\ln p_{\boldsymbol{\theta}}(\mathbf{x}) = D_{\text{KL}}(q_{\boldsymbol{\phi}}(\mathbf{z}|\mathbf{x})||p_{\boldsymbol{\theta}}(\mathbf{z}|\mathbf{x})) + \mathbb{E}_{q_{\boldsymbol{\phi}}(\mathbf{z}|\mathbf{x})}[\ln p_{\boldsymbol{\theta}}(\mathbf{x}|\mathbf{z})] - D_{\text{KL}}(q_{\boldsymbol{\phi}}(\mathbf{z}|\mathbf{x})||p(\mathbf{z})). \tag{4}$$

The penultimate term encodes our first goal; and the final term, our second goal.

Recall that the Kullback-Leibler divergence is nonnegative. The sum of the final two terms therefore lower-bounds the log-likelihood, $\ln p_{\boldsymbol{\theta}}(\mathbf{x})$. $\mathbf{x}$ denotes the event observed, $\boldsymbol{\theta}$ denotes a possible cause, and $p_{\boldsymbol{\theta}}$ denotes the likelihood that $\boldsymbol{\theta}$ caused

$\mathbf{x}$. Maximizing each side of Eq. (4), and invoking Ineq. (3), yields

$$\max_{\theta}\{\ln p_{\boldsymbol{\theta}}(\mathbf{x})\} \geq \max_{\theta}\left\{\mathbb{E}_{q_{\boldsymbol{\phi}}(\mathbf{z}|\mathbf{x})}\left[\ln p_{\boldsymbol{\theta}}(\mathbf{x}|\mathbf{z})\right] - D_{\mathrm{KL}}\big(q_{\boldsymbol{\phi}}(\mathbf{z}|\mathbf{x})||p(\mathbf{z})\big)\right\}. \tag{5}$$

The RHS is called the *evidence lower bound* (ELBO).

A variational autoencoder is a neural network that implements the ELBO. $q_{\boldsymbol{\phi}}(\mathbf{z}|\mathbf{x})$ encodes the input $\mathbf{X}$, and $p_{\boldsymbol{\theta}}(\mathbf{x}|\mathbf{z})$ decodes. The variational autoencoder has the cost function

$$\mathscr{L}_{\mathrm{VAE}} := \mathbb{E}_{p_{\mathrm{emp}}(\mathbf{x})}\left[\mathbb{E}_{q_{\boldsymbol{\phi}}(\mathbf{z}|\mathbf{x})}\left[\ln p_{\boldsymbol{\theta}}(\mathbf{x}|\mathbf{z})\right] - D_{\mathrm{KL}}\big(q_{\boldsymbol{\phi}}(\mathbf{z}|\mathbf{x})\|p(\mathbf{z})\big)\right]. \tag{6}$$

$p_{\mathrm{emp}}(\mathbf{x})$ denotes the distribution inferred from the empirical dataset. Given input values $\mathbf{x}$, the variational autoencoder generates a latent distribution $q_{\boldsymbol{\phi}}(\mathbf{z}|\mathbf{x}) = \mathscr{N}(\boldsymbol{\mu}_{\mathbf{z}|\mathbf{x}}, \boldsymbol{\Sigma}_{\mathbf{z}|\mathbf{x}})$. We denote by $\mathscr{N}(\boldsymbol{\mu}, \boldsymbol{\Sigma})$ the standard multivariate normal distribution whose vector of means is $\boldsymbol{\mu}$ and whose covariance matrix is $\boldsymbol{\Sigma}$. Neural-network layers parameterize the variational autoencoder's $\boldsymbol{\mu}_{\mathbf{z}|\mathbf{x}}$ and $\boldsymbol{\Sigma}_{\mathbf{z}|\mathbf{x}}$. Latent vectors are sampled according to $q_{\boldsymbol{\phi}}(\mathbf{z}|\mathbf{x})$, then decoded into outputs distributed according to $p_{\boldsymbol{\theta}}(\mathbf{x}|\mathbf{z}) = \mathscr{N}(\boldsymbol{\mu}_{\mathbf{x}|\mathbf{z}}, \sigma^2_{\mathbf{x}|\mathbf{z}}\mathbb{1})$. Neural-network layers parameterize the mean vector $\boldsymbol{\mu}_{\mathbf{x}|\mathbf{z}}$. The variance $\sigma^2_{\mathbf{x}|\mathbf{z}}$ is a hyperparameter.

A variational autoencoder with the following architecture produced the results in the main text. The style was borrowed from[4]. Two fully connected 200-neuron hidden layers process the input data. One fully connected two-neuron hidden layer parameterizes each of $\boldsymbol{\mu}_{\mathbf{z}|\mathbf{x}}$ and $\boldsymbol{\Sigma}_{\mathbf{z}|\mathbf{x}}$. Two fully connected 200-neuron hidden layers process the latent variables. An output layer reads off the outputs. We choose $\sigma^2_{\mathbf{x}|\mathbf{z}} = 1$ and use Rectified Linear Unit (ReLU) activations for all hidden layers.

II Justification of use of machine learning

Deep learning is a powerful tool. Is it necessary for recovering our results? Could simpler algorithms detect and quantify many-body learning as sensitively? Comparable simpler algorithms tend not to, we find. Two competitors suggest themselves: single-layer linear autoencoders, related to principal-component analysis[5], and clustering algorithms. Alternatives include generalized linear models[6] and supervised linear autoencoders. These models, however, perform supervised learning. They receive more information than the variational autoencoder and so enjoy an unfair advantage. Furthermore, supervised learners receive information not directly available to the many-body system—the drives' labels (see Results). One therefore cannot infer, from a supervised learner's output, how much the many-body system has learned. We analyze the two comparable competitors sequentially.[1]

II A Comparison with single-layer linear autoencoder

The linear autoencoder is a single-layer neural network. The input, X, undergoes a linear transformation: $Y = mX + b$. We compare, as follows, the linear autoencoder's detection of field classification with the variational autoencoder's detection: We trained the spin glass on a drive in each of 3,000-5,000 trials. Ninety percent of the trials were designated as neural-network-training data; and 10%, as neural-network-testing data. For each training trial, we identified the spin glass's final configuration. On these configurations, each neural network performed unsupervised learning. Each neural network then received the configuration with which the spin glass ended a neural-network-testing trial. We inferred the field most likely to have produced this configuration, using maximum-likelihood estimation. The fraction of trials in which the neural network points to the correct field constitutes the neural network's score. On a three-field drive, the linear autoencoder scored 0.771, while the variational autoencoder scored 0.992. On a five-field drive, the linear autoencoder scored 0.3934, while the variational autoencoder scored 0.829. Hence the variational autoencoder picks up on more of the spin glass's ability to classify fields.

II B Comparison with clustering algorithm

A popular, straightforward-to-apply algorithm is *k-means clustering*[6]. k refers to a parameter inputted into the algorithm, the number of clusters expected in the data. We inputted the number of drives imposed on the spin glass, in addition to inputting configurations. The variational autoencoder receives just configurations and so less information. We could level the playing field by automating the choice of k, using the Bayesian information criterion (BIC)[6]. But clustering with the BIC-chosen k would perform no better than clustering performed with the ideal k, and the ideal clustering performs worse than the variational autoencoder.

The protocol run on the spin glass is described at the beginning of Methods, in the memory-capacity study. Five thousand trials were performed. The configuration occupied by the spin glass at the end of each trial was collected. Splitting the data

[1] Alternative representations of inputs have been defined to facilitate predictions, e.g.,[7]. We seek a representation (e.g., a latent space) that elucidates how the spin glass's configurations reflect the drive. This problem differs from that addressed in[7]. Nevertheless, modifications of recent techniques of Crutchfield *et al.* may provide useful alternatives to the standard machine-learning toolkit in future work.

into testing and training data did not alter results significantly. Hence we fed all the configurations, with the number $k = 5$ of drives, to the clustering algorithm. The algorithm partitioned the set of configurations into subsets. Each subset contained configurations likely to have resulted from the same drive.

Clustering algorithms are assessed with the Rand index, denoted by RI[8]. The Rand index differs from the maximum-likelihood-estimation score (discussed in the Results). How to compare the clustering algorithm with the variational autoencoder, therefore, is ambiguous. However, the Rand index quantifies the percentage of the algorithm's classifications that are correct. Hence the Rand index and the maximum-likelihood-estimation score have similar interpretations, despite their different definitions.

The clustering algorithm's Rand index began at $\text{RI} = 0$, at $t = 0$. RI rose during the first ≈ 200 changes of the drive, then oscillated around 0.125. Figure 6 shows the variational autoencoder's performance. The variational autoencoder's score rose during the first ≈ 150 changes of the drive, then oscillated around $0.450 > 0.125$. Hence the variational autoencoder outperformed the clustering algorithm.

III Distinction between robust learning and two superficially similar behaviors

Learning contrasts with two other behaviors that the spin glass could exhibit, entraining to the field and near-freezing.

III A Entraining to the field

Imagine that most spins align with any field A. The configuration reflects the field as silly putty reflects the print of a thumb pressing on the silly putty. Smoothing the silly putty's surface wipes the thumbprint off. Similarly, applying a field $B \neq A$ to the spin glass wipes the signature of A from the configuration. From the perspective of the end of the application of B, the spin glass has not learned A. The spin glass lacks a long-term memory of the field; the field is encoded in no robust, deep properties of the configuration.

We can distinguish learning from entraining by calculating the percentage of the spins that align with the field at the end of training. If the spins obeyed the field, 100% would align. If the spins ignored the field, 50% would align, on average. Hence the spin glass's entraining is quantified with

$$2(\text{percentage of spins aligned with the field}) - 100. \tag{7}$$

(This measure does not apply to alignment percentages < 50, which are unlikely to be realized.)

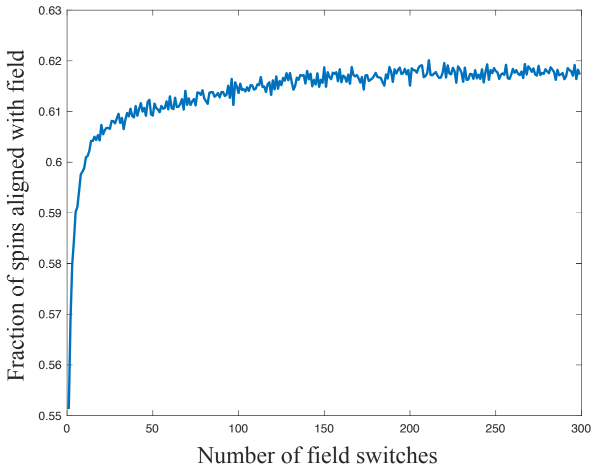

Figure 1. Fraction of the spins aligned with the field, as a function of time: If a fraction ≈ 1 of the spins align, the spin glass resembles silly putty, which shallowly reflects the print of a thumb that presses on it. Robust learning stores information deep in a system's structure.

Figure 1 shows data collected about the spin glass in the good-learning regime (introduced in the "Spin glass" section of the Results). The number of aligned spins is plotted against the amount t of time for which the spin glass has trained. After the application of one field, 55% of the spins align with the field. At the end of training, 62% align. Hence the spins' entraining grows from 10% to 24%. Growth is expected, as the spin glass learns the training drive. But 24% is an order of magnitude less than 100%, so the spin glass is not entrained to the field.

III B Near-freezing

Suppose that the spin glass is nearly frozen. Most spins cannot flip, but a few jiggle under most fields. The spin glass does not learn any field effectively, being mostly immobile. But the few flippable spins reflect the field. A bottleneck neural network could guess the field from those few spins. The neural network's low loss function would induce a false positive, leading us to believe that the spin glass had learned.

We can avoid false positives by measuring two properties. First, we measure the percentage of the spins that antialign with the field. If the percentage consistently $\gg 0$, many of the spins are not frozen. Figure 1 confirms that many are not.

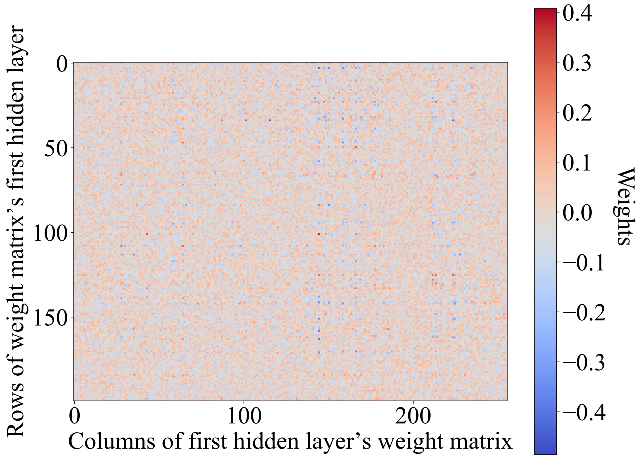

Figure 2. How much information about each spin the variational autoencoder compresses: This figure represents the first hidden layer's weight matrix. The weight matrix transforms the input layer, which consists of 256 neurons, into the first hidden layer, which consists of 200 neurons. The matrix's elements are replaced with colors. Each vertical line corresponds to one spin. The farther leftward a stripe, the lesser the spin's field energy [Eq. (1)].

Second, we check that the neural network compresses information about spins that have many different field energies $A_j(t)s_j$ [Eq. (1)]. We illustrate with the protocol used to generate Fig. 2: We trained the spin glass on a drive $\{A, B, C\}$ in each of many trials. On the end-of-trial configurations, the neural network was trained.

A configuration is represented in the neural network's input layer, a column vector. A weight matrix transforms the input layer into the first hidden layer, another column vector. The weight matrix is depicted in Fig. 2. The matrix's numerical entries have been replaced with colors. Each vertical stripe corresponds to one spin. The farther leftward a stripe, the lesser the spin's field energy. The darker a stripe, the more information about the spin the neural network uses when forming Z. The plot is approximately invariant, at a coarse-grained level, under translations along the horizontal. (On the order of ten exceptions exist. These vertical stripes contain several dark dots. An example appears at $x \approx 150$. But the number of exceptions is much less than the number of spins: $\approx 10 \ll 256$.) Hence the neural network uses information about spins of many field energies. The spins do not separate into low-field-energy flippable spins and high-field-energy frozen spins.

IV Memory capacity attributed to the many-body system by the absorbed power

In the Methods, we compared the memory capacity registered by the neural network to the capacity registered by the absorbed power. The study involved maximum-likelihood estimation on drives of 40 fields selected from 50 fields. The choice of 50 is explained here: Fifty fields exceed the spin-glass capacity registered by the absorbed power.

Recall how memory has been detected thermodynamically[9]: Let a many-body system be trained with a drive that includes a field A. Consider testing the system, afterward, with an unfamiliar field B, and then with A. Suppose that the absorbed power jumps substantially when B is applied and less when A is reapplied. The many-body system identifies B as novel and remembers A, according to the absorbed power.

We sharpen this analysis. First, we divide the trial into time windows. During each time window, the field switches 10 times. (The 10 eliminates artificial noise and is not critical. Our qualitative results are robust with respect to changes in such details.) We measure the absorbed power at the end of each time window and at the start of the subsequent window. We define "the absorbed power jumps substantially" as "the absorbed power jumps, on average over trials, by much more than the noise

(by much more than the absorbed power fluctuates across a trial)":

$$\langle(\text{Power absorbed at start of later time window}) - (\text{Power absorbed at end of preceding time window})\rangle_{\text{trials}} \tag{8}$$
$$\gg \text{Standard deviation in } [(\text{Power absorbed at start of later window}) - (\text{Power absorbed at end of preceding window})].$$

Consider including only a few fields in the training drive, then growing the drive in later trials. The drive will tax the spin glass's memory until exceeding the capacity. The LHS of (8) will come to about equal the RHS.

Figure 3 illustrates with the spin glass. On the x-axis is the number of fields in the training drive. On the y-axis is the ratio of the left-hand side of Ineq. (8) to the right-hand side (LHS/RHS). Where LHS/RHS ≈ 1, the spin glass reaches its capacity. This spin glass can remember ≈ 15 fields, according to the absorbed power.

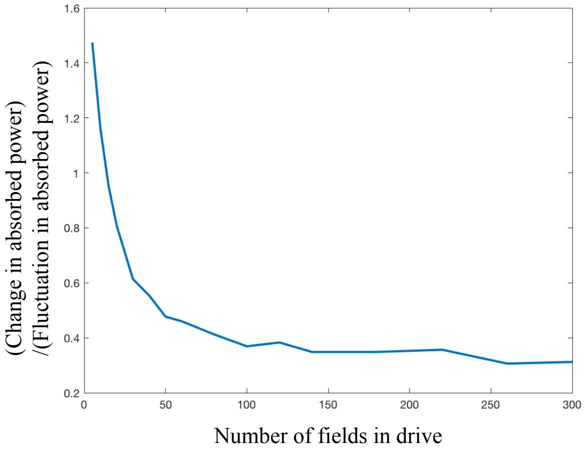

Figure 3. Estimate of memory capacity by absorbed power: A many-body system reaches its capacity, according to the absorbed power, when [left-hand side of Ineq. (8)] / (right-hand side) ≈ 1. The curve ≈ 1, and a 256-spin glass reaches its capacity, when the training drive contains ≈ 15 fields.

References

1. Kingma, D. P. & Welling, M. Auto-Encoding Variational Bayes. *arXiv:1312.6114* (2013).
2. Jimenez Rezende, D., Mohamed, S. & Wierstra, D. Stochastic Backpropagation and Approximate Inference in Deep Generative Models. In *Proc. 31st Int. Conf. on Machine Learning* (2014).
3. Doersch, C. Tutorial on Variational Autoencoders. *arXiv:1606.05908* (2016).
4. Hafner, D. Building variational auto-encoders in tensorflow. Blog post (2018).
5. Bourlard, H. & Kamp, Y. Auto-association by multilayer perceptrons and singular value decomposition. *Biol. Cybern.* **59**, 291–294, DOI: 10.1007/BF00332918 (1988).
6. Bishop, C. M. *Pattern Recognition and Machine Learning* (Springer, 2006).
7. Shalizi, C. R. & Crutchfield, J. P. Computational mechanics: Pattern and prediction, structure and simplicity. *J. Stat. Phys.* **104**, 817–879, DOI: 10.1023/A:1010388907793 (2001).
8. Rand, W. M. Objective criteria for the evaluation of clustering methods. *J. Am. Stat. Assoc.* **66**, 846–850, DOI: 10.1080/01621459.1971.10482356 (1971). https://www.tandfonline.com/doi/pdf/10.1080/01621459.1971.10482356.
9. Gold, J. M. & England, J. L. Self-organized novelty detection in driven spin glasses. *arXiv:1911.07216* (2019).
